# Technological progress, globalization and low-inflation: Evidence from the United States

**DOI:** 10.1371/journal.pone.0215366

**Published:** 2019-04-18

**Authors:** Lei Lv, Zhixin Liu, Yingying Xu

**Affiliations:** 1 School of Economics and Management, Beihang University, Beijing, China; 2 University of Science & Technology Beijing, Beijing, China; Universitat de Valencia, SPAIN

## Abstract

Since the late 1990s, particularly since the global financial crisis, the core inflation of main developed economies’ has been persistently below target. The factors hindering the achievement of inflation targets are nothing more than commodity price, oil supply, weakness of aggregate demand, and various other factors. In addition, technology and globalization have also played a significant role. This paper uses an extended hybrid New Keynesian Phillips Curve (NKPC) model to quantify the contribution of technology and globalization variables to inflation in the United States (U.S.). The analysis suggests that technology and globalization well explain the low inflation dynamics in the U.S., as the impact of globalization on domestic inflation has been weakening over the past 20 years or so, while the impact of technology on inflation has been increasing. At present, technology exerts a greater role than globalization on low-inflation in the U.S.. This raises a different perspective for understanding the phenomenon of low inflation in the U.S. and other regions.

## 1. Introduction

The Great Recession of 2008–2009 was one of the most severe recessions in decades, and its impact on inflation dynamics in various countries has been widely researched but is not yet fully understood. At present, the global economy is either at or near full employment, but inflation remains in the doldrums. Nevertheless, a decade after the outbreak of the global financial crisis, the central banks of the U.S., Europe, Japan, and other regions still strive to achieve their 2% inflation goals despite years of extraordinary stimulus measures. In particular, inflation in the U.S. has been running at low levels. Over the five years ending in December 2017, the percent change in the Consumer Price Index (CPI), at 7.25% (or 1.41% at an annual rate), was the lowest rate of price increase seen in the U.S. in half a century [1]. As the Federal Reserve System (FED) Chairman Jerome Powell said at Jackson Hole conference in 2018:“Inflation has moved up and is now near the Federal Open Market Committee's (FOMC) objective of 2% after running generally below that level for six years. While inflation has recently moved up near 2%, we have seen no clear sign of an acceleration above 2%, and there does not seem to be an elevated risk of overheating” [2]. The causes and potential consequences of low inflation have been an area of intense research [3–6].

This paper looks at low inflation in the U.S., with a special focus on the 1999–2016 period. It discusses some of the structural and cyclical factors behind inflation developments and proposes answers to two main questions: (i) What is the status of low inflation in the U.S.? (ii) What is the reason for the low inflation in the U.S.?

China’s slowdown, demographics, globalization, and ‘transitory factors’ are commonly cited reasons for the common low inflation phenomenon. Whether the devaluation of the Ren Min Bi has made Chinese products cheaper, falling commodity prices, or demand in the global economy has fallen, the slowdown in China’s economy will weaken inflationary pressures and raise concerns about global deflation spirals (International Monetary Fund, 2016). Demographic change is also often mentioned in this respect. This certainly heralds important economic shifts, but its impact on inflation is ex ante unclear. If aggregate demand is lower than aggregate supply, demographic change may put downward pressure on prices. But demographic change can also create impetus for price increases: according to the life-cycle hypothesis, an aging population means that the elderly eventually dissave and consume more [7]. Many observers believe that globalization of the economy has changed the behavior of inflation. Greenspan states that globalization “appears to be an essential element of any paradigm that can explain the events of the past decade”, including low inflation [8]. The financial markets including insurance markets are also significantly affected by the globalizations [9]. The Economist (2005) suggests that increased trade makes a mockery of traditional economic models of inflation, which generally ignore globalization [10]. The Globalization Hypothesis argues that the internationalization of goods and financial markets has already affected the domestic macroeconomic determinants, such as inflation rate and business cycle, through international influence [11].

The U.S. is quite an open economy today, and its trading partner’ economic performance plays an important role in the slowly solidifying recent cyclical recovery [12]. According to Bernanke [13], increased trade with China and other developing countries has led to slower growth in the prices of imported manufactured goods. He cited a study concluding that trade with China alone reduced annual import price inflation in the U.S. by approximately 1% over 1993–2002. On the other hand, as technology is more prominently used to produce more goods and services, companies in all industries are achieving lower production costs. Since 2001, the prices of computers and electronic products, computer designs, and computer services have been declining, and other technology inputs have been causing the production cost and the final product price to drop by 0.5% each year [14]. According to data from the National Bureau of Statistics in 2017, telecom service provider Verizon’s decision to provide unlimited data packages has brought down the U.S. core inflation rate by 0.2% in June. Technological innovation has had a negative impact on inflation in many dimensions.

Regarding whether globalization elements should be included within the scope of domestic inflation drivers, the debate in academic circles has been fierce in recent years, and existing research is far from consensus. For example, Tootell [15] uses the output gaps of the six major trading partners of the U.S. as indicators of globalization, and uses 1973–1996 data to estimate the U.S.’s traditional Phillips curve model. The study did not find evidence that globalization affects the U.S. domestic inflation rate. Gamber & Hung [16] also study the effects of globalization on U.S. inflation in the framework of the traditional Phillips Curve model, but they increase the number of major trading partners considered to 35 and update the end of the sample interval to 1999. The results show that the globalization factor has a positive effect on U.S. inflation, but only at the 10% significance level. If the trading partner country changed to Organization for Economic Cooperation and Development (OECD) countries, the effect of the globalization indicator variable (measured by the foreign output gap) on U.S. inflation was no longer significant at this time [17]. Given the weak empirical evidence based on the U.S., Ball [18] believes that globalization is difficult to generate for domestic inflation. The reason for the significant impact is that the pricing of domestic companies is mainly affected by the domestic excess demand on their marginal costs and has little relevance to the international market. Calza [19] also comes to similar conclusions when testing whether the proposition that globalization has led to greater sensitivity of domestic inflation to the global output gap holds for the euro area.

However, several other empirical studies have confirmed the existence of the relationship between globalization and inflation. Borio & Filardo [20] provide evidence supporting the impact of globalization on inflation in OECD countries. They estimate that the weighted average foreign output gap has a significant positive effect on domestic inflation, and it has shown an upward trend year by year. Milani [21] explores the effect of increasing globalization on the dynamics of macroeconomic variables in the U.S. and find that sensitivity of inflation in the U.S. to global output indicators has increased over time, albeit with only minor changes. Chang [22] investigates the causal linkages between globalization and inflation in 21 OECD countries by using panel causality analysis for 1970–2010. The study conclusions indicate that globalization has significantly changed some major industrialized countries’ inflation and show the effect of globalization on the inflation exhibits a high degree of heterogeneity.

These studies confirm that globalization has an impact on inflation, and some studies propose that the impact of a global output gap on domestic inflation has exceeded the impact of a domestic output gap on inflation. Manopimoke [23] finds that a global output gap has replaced the domestic output gap as the key driving variable for inflation in 17 advanced and emerging countries, particularly since the year 2000. Zhang et al. [24] evaluate whether globalization has increased the role of global factors in driving inflation in China. Empirical results show that the global output gap significantly affects the dynamics of inflation in China. In particular, the global output gap is superior to the domestic output gap in predicting domestic inflation. But there have also been opposing conclusions. Using a time-varying VAR, Bianchi and Civelli [25] investigate whether global economic slack has progressively replaced the domestic output gap in driving inflation as globalization increases. They conclude indicate that integration in the global economy is in fact important, but globalization has not yet induced changes in openness large enough to justify significant brakes in inflation dynamics.

In addition to these factors, technology is another important and often overlooked factor that explains why inflation has tended to fall short of the 2% targets by as much as it has. Alan Greenspan stated in testimony before the U.S. Congress in 2005: “The past decade of low inflation and solid economic growth in the United States and in many other countries around the world … is attributable to the remarkable confluence of innovations that spawned new computer, telecommunication, and networking technologies, which, especially in the United States, have elevated the growth of productivity, suppressed unit labor costs, and helped to contain inflationary pressures” [26]. His idea, echoing the voices of many other economists and observers, is that technological advancement has brought down the price of goods that use new technologies intensively. Coined by the Intel co-founder Gordon Moore, Moore's Law has become synonymous with more powerful and cheaper technologies [14]. Moore’s Law is the observation that the number of transistors in a dense integrated circuit doubles about every two years. As technology continues to improve, the relative price of technology continues to decline. In recent years, many scholars have postulated that the possible disinflationary effects of technical application (such as digitalization and e-commerce) could explain the subdued inflation in advanced and emerging market economies. Scholars' research about the impact of technology on inflation is mainly divided into the following three aspects:

Technological innovation has a direct impact on the changes in the prices of information and communication technologies, leading to continued decline in the prices of computers and home electronics [27, 28]. The price of some information and communications technology (ICT) products has rapidly decreased from the 1990s onward, due to technological change [29]. Masse and Beaudry [27] study the contribution of ICT products to inflation in Canada. They find that the level of competition in the industry is much lower than in other countries, but the price of communications has not declined in Canada. Since the price component of ICT is heavily weighted in the CPI, its price changes dominate other ICT components. Therefore, the contribution of ICT products (communications and IT) to CPI growth has occasionally been negative in Canada. Moreover, as products become more specialized, it becomes more difficult to measure price changes. Similarly, more and more free digital products (such as applications and online travel bookings) are neither well-captured in nominal gross domestic product (GDP) nor recorded in the CPI [30].

Technology will have an impact on competition and market structure. It will reduce the barriers for new company creation in many areas, exacerbate market competition, and, thus, affect product price [31, 32]. Blix [31] show that the rapid growth of e-commerce is another way by which digitalization can increase competitiveness and influence inflation. New technologies have changed the way that consumers search for and compare product prices, and these customers benefit from increased price transparency and comparability. Yi and Choi [33] tested the impact of e-commerce on inflation using cross-country panel data from 1991 to 2000. The results show that the internet improves productivity and, thus, will reduce inflation. Almost any company (whether large or small or start-up) can now go global and reach potential customers faster and at lower cost. In addition, competition from digital firms is invading non-tech sectors, and foreign competition is reaching domestic markets more easily, which may create competitive low prices in local retail [34]. On the other hand, trade openness plays an important role in attracting foreign direct investment, which is likely to affect the globalization and technology progress in particularly the trading countries such as Brazil, Russia, India, China, and South Africa (BRICS) and Mexico, Indonesia, Nigeria, and Turkey (MINT) [35].

Technology also increases productivity, lowers the rate of wage growth relative to productivity, and then delays rising inflation [32, 34]. Technology innovation serves as a complement to the workforce, and the impact of deflation is straightforward. Higher productivity translates directly into lower production costs. If Policy-makers can tolerate temporary low inflation without any reaction, the price level will be permanently lower, with no long-term impact on inflation [34]. Technology affects inflation by creating productivity improvements through the substitution of labor by, for example, automation. The increase in total demand is suppressed due to the artificial replacement, which may lead to deflationary effects. Autor et al. [36] has found a strong link between the fall of the labor share income and the rise of superstar firms in the U.S. As wealthy peoples’ marginal propensity to consume is lower, worsening income distribution may continue to drag down aggregate demand and may curb rising inflation.

In summary, globalization and technology are vised as important factors affecting inflation, but tested separately. Furthermore, the research literature on the impact of technology on inflation is usually based on descriptive and commentary analysis rather than quantitative estimation [14, 27, 31, 32, 34, 35]. This paper intends to consider the impacts of globalization and technology on inflation simultaneously. We add the technology variable and globalization variable into a model (hybrid extended New Keynesian Phillips Curve) for empirical analysis to quantify the role played by them in driving inflation in the U.S. The results help us understand the recent low inflation in the U.S. and benefits the future management of inflation.

The rest of the paper is organized as follows. Section 2 outlines the research methodology. Section 3 describes the data used in empirical work and some stylized facts in the United States. Section 4 presents the empirical findings of this research and our discussion. Section 5 concludes the paper.

## 2. Empirical methods

The Phillips curve can be broadly described as the relationship between inflation and economic slack and extends to other factors that affect price changes. In the new Keynesian framework, those factors specifically include inflation expectations. This relationship is called the New Keynesian Phillips Curve (NKPC) [37]. It assumes that only a fraction of the price (1−*α*,0<*α*<1) is adjusted during each period, while the rest (*α*) remains unchanged [38]. Therefore, the current inflation rate (*π*_*t*_) is a function of the current expected price change (*E*_*t*_*π*_*t*+1_) and is usually seen as rational and is a cyclical component of economic activity (${\hat{y}}_{t}$), the deviation of output or real marginal costs from their trends:

$$\pi_{t}=\beta E_{t}\pi_{t+1}+k{\hat{y}}_{t}+\varepsilon_{t}$$ (1)

where *ε*_*t*_ is a random disturbance term with independent consistency distribution characteristics. From an empirical perspective, there are some doubts about the NKPC. This relationship can neither explain the phenomenon of persistently high inflation nor predict the cost of deflation in the real economy [39]. This inconsistent experience leads to an alternative specification of the above relationship, namely the Hybrid New Keynesian Phillips Curve, in which the explanatory variable also contains lagging inflation:

$$\pi_{t}=\omega E_{t}\pi_{t+1}+(1-\omega )\pi_{t-1}+k{\hat{y}}_{t}+\varepsilon_{t}$$ (2)

Among them, the coefficient 1−ω measures the degree of influence of past inflation on current inflation, which is the intensity of inflation inertia; and ω measures the degree of influence of future inflation expectations on current inflation, that is the intensity of inflation expectations. By Galí and Gertler [40], Galí et al. [41, 42] representative research suggests that inflation expectations should dominate in the HNKPC model, that means the value of 1−ω is much larger than the value of ω.

Our empirical specification is based on Bianchi and Civelli, Zhang and He [25, 43], who estimate an extended hybrid NKPC (HNKPC) for study globalization and inflation, in which the globalization indicator is represented by foreign output gap. The theoretical model (2) implies that HNKPC is stable in the short term, and inflation varies along a fixed HNKPC, without considering that HNKPC may move under various shocks. In order to characterize the effects of various shocks, we introduce other exogenous variables (such as technical shock, federal funds rate shock, exchange rate shock and unit labor costs) [26, 44–46] and random variables in the empirical model. Therefore, the model based on model (2) can be specified as:

$$\pi_{t}=c+\gamma_{e}E_{t}\pi_{t+1}+\gamma_{b}\pi_{t-1}+\delta_{d}y_{t}^{d}+\delta_{f}y_{t}^{f}+\phi_{g}tech_{t}+X_{t}\beta +\eta_{t}$$ (3)

where *π*_*t*_ is the current inflation rate, *E*_*t*_*π*_*t*+1_ denotes the currently expected price changes, *π*_*t*−1_ is the lagged inflation, $y_{t}^{d}$ is the domestic output gap, and $y_{t}^{f}$ denotes the weighted foreign real output gap, which represents the globalization, *tech*_*t*_ is the drag of technology on the inflation, also is the variable of interest in this article. Technology affects prices via sectoral innovation and unit labor costs [26]. The tech variable can be capturing the first component (and it is strong enough to lower overall inflation), while the second component (unit labor costs) as a control variable in our analysis. *X*_*t*_ are the control variable, other factors that affect inflation. This paper selects the federal funds rate *γ*_*t*_ denotes monetary policy shock, the U.S. dollar index *USDX*_*t*_ denotes exchange rate shock and the nonfarm labor productivity *NLP*_*t*_ denotes unit labor costs. *η*_*t*_ is a random disturbance term with independent consistency distribution characteristics.

This study adopts the extended HNKPC to examine the driving factors in determinants of low inflation in the United States. We estimate the model using Generalized Method of Moments (GMM).

## 3. Data

The baseline estimation of model (3) involves series for inflation *π*_*t*_, inflation expectations *E*_*t*_*π*_*t*+1_, domestic output gap $y_{t}^{d}$, a measure of the foreign real output gap $y_{t}^{f}$, the drag of the technology variable *tech*_*t*_, U.S. dollar index *USDX*_*t*_, federal funds rate *r*_*t*_ and nonfarm labor productivity *NLP*_*t*_. We use quarterly U.S. data spanning 18 years from 1999:Q1 to 2016:Q4, dictated by the availability of quarterly trade data.

Inflation is measured using the personal consumption expenditures (PCE) excluding food and energy (chain-type price index). Looking at core indexes, rather than focusing on a short episode of spikes in inflation, helps to observe the inflation trend [26]. The comparatively less fluctuating property of core inflation is more applicable in testing the effects of globalization and technology on inflation [47–49]. Meanwhile, the core inflation has been an important index that attracts attention of many currency governments such as Federal Reserve [50], the European Central Bank [51], and the Bank of Japan [52]. Survey forecasts of inflation are taken from the Survey of Professional Forecasters. We consider one-year-ahead inflation forecasts made at time t, *E*_*t*_*π*_*t*+1_. The domestic output gap equals the difference between either the actual or the projected GDP and the Congressional Budget Office’s estimate of potential GDP. The inflation series, Federal Funds Rate, U.S. dollar index quarterly data and the nonfarm labor productivity quarterly data were from the St. Louis Fed's FRED database. The Survey of Professional Forecasters and the domestic output gap series were from the Federal Reserve Bank of Philadelphia’s database and the Congressional Budget Office’s website, respectively.

**Fig 1:**
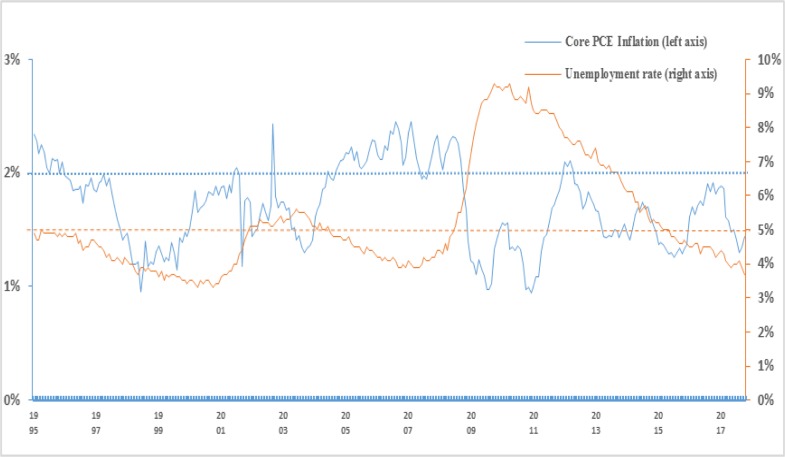
U.S. core inflation. ***Note*:** Percent change is the change over the previous one year. The data shown in the figure span the period from January 1999 to December 2017. ***Source*:** Federal Reserve Bank of St. Louis economic database.

Generally, the core inflation in the U.S. has been persistently below target since 2009, but the unemployment rates decreased from 7.7% (in 2010) to 4.3% (2017), as shown in **Fig 1**. As can be seen from the figure, before 2010, there was a negative correlation between core inflation and the unemployment rate basically, which means that a lower core inflation is accompanied with a higher unemployment rate, agreeing with the Phillips curve theory. Data after 2010 show that this negative correlation is no longer obvious. Specifically, the unemployment rate was constantly decreasing, but the core inflation rate maintained around 2%. The FED defined its monetary policy goal as full employment and the FOMC's 2% long-run objective since that goal was announced in January 2012 [5]. The decreasing unemployment rate indicates that the U.S. labor market has recovered strongly and inflation is close to the FED's 2% target, which creates a sufficient policy space for the Fed's future projects. In this paper, we adopt the models based on NKPC to estimate the factors driving the dynamics of core inflation.

The drag of the technology variable and the foreign output gap are calculated according to Davis [14]. Meanwhile, Dewett and Jones's [53] review paper made a detailed review on the information technology (IT) including 37 papers within the last five years that have been published in six leading management journals. According to the type of IT summarized in the literature, we identify technology-related inputs in the I-O data as follows: computer and electronic products *tech*_1_; broadcasting and telecommunications *tech*_2_; data processing, internet publishing and other information services *tech*_3_; and computer systems design and related services *tech*_4_. The producer price index (PPI) series for each industry in the I-O, including all four technical inputs $ppi_{tech_{\kappa }}$, where $ppi_{tech}=\sum ppi_{tech_{\kappa }}$ and *κ* = 1,2,3,4 are from the Bureau of Labor Statistics Producer Price Indexes Databases. As for the contribution of technology price to price increases in various industries, the weights of technical inputs are calculated using the prices in each year, $W_{tech_{\kappa }}=tech\;input_{\kappa }/all\;input$ (quarterly observations within one year use the same weight of the year). These weights are multiplied by the technology's PPI to derive the technology's contribution to each industry's PPI. Thus, the technology variable is $tech={\sum W_{tech_{\kappa }}\times ppi}_{tech_{k}}$. Industries are defined according to the 2007 North American Industry Classification System.

We calculate foreign output gaps according to [19, 25, 54]. The weighted average of the output gaps of the major trading partners of the countries studied is used as the country’s foreign output gap. The study of [54] computed China’s foreign output gap by aggregating data on China’s top 18 major trading partners using trade weights derived from bilateral trade statistics. The weight for each trade partner in each year is determined by the percentage of the partner’s trade (both exports and imports) to China over the total trade between China and the 18 partners for that year. Then foreign output gap is calculated by $y_{t}^{f}=\sum_{j=1}^{18}w_{j,t}y_{j,t}$, where *w*_*j*,*t*_ denotes the defined weight (i.e. trade percentage) at time t (quarterly observations within one year use the same weight of the year) and *y*_*j*,*t*_ is the output gap measure for country/region j.

In this paper, we choose U.S.’s 13 major trading partners by the trade data obtained from the U.S Bureau of Economic Analysis database. The foreign real output gap is calculated by $y_{t}^{f}=\sum_{j=1}^{13}w_{j,t}y_{j,t}$, where *w*_*j*,*t*_ denotes the defined weight (i.e. trade percentage) at time t (quarterly observations within one year use the same weight of the year) and *y*_*j*,*t*_ is the output gap measure for country/region j. The total import and export volume of these 13 trading partners accounts for 67%~73% of the total import and export volume of the U.S. during the period from 1999 to 2016.

**Fig 2:**
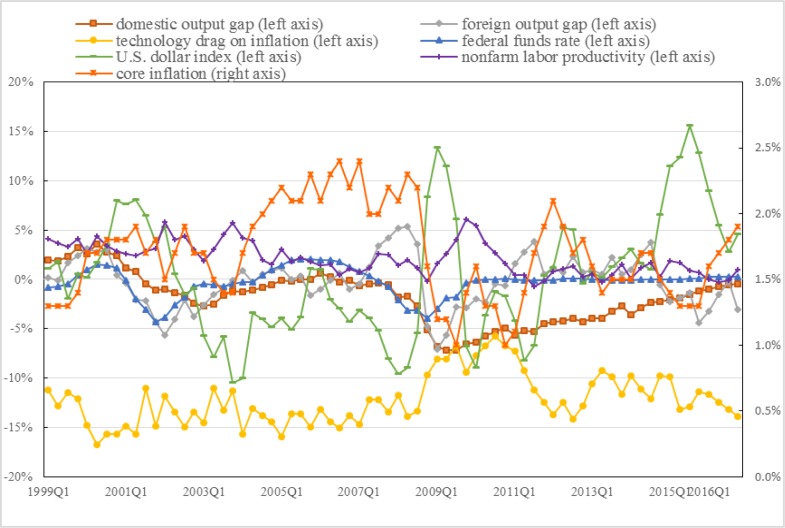
U.S. core inflation, domestic and foreign output gap, technology drag, federal funds rate, U.S. dollar index and nonfarm labor productivity: 1999–2016. ***Note*:** Data calculated by the author.

Based on the above description, **Fig 2** depicts the dynamic timing diagram of the core inflation rate, domestic and foreign output gaps, technical variables, federal funds rate, U.S. dollar index and nonfarm labor productivity in the U.S. from 1999:Q1 to 2016:Q4. As can be seen from the figure, the domestic and foreign output gaps have a certain degree of periodicity in the overall change trend before 2009:Q1, but there are obvious differences in the specific change trends at each time point, especially the peaks. The locations and times of the troughs are different. Starting from 2009:Q1, the domestic output gap steadily decreases, while the foreign output gap fluctuate around the zero-bound value. It is worth paying special attention to the fact that the cyclical trend of the foreign output gap has a strong consistency with the core inflation in the United States. The foreign output gap in 2002 and 2009 coincides exactly with the lowest point of the core inflation rate in these periods, and the crest point is also in line with the peak of core inflation in 2008 and 2012. Compared with the foreign output gap, the domestic output gap appears smoother, and its cyclical changes are low, similar to core inflation trends. There are two reasons why the domestic output gap appears smoother. First, according to the findings of [55], the U.S., the United Kingdom, Canada, and Australia in which the financial system is dominated by the capital market can always recover from financial crises faster than other countries studied. In [55], two IMF economists studied the economic developments of 17 OECD developed countries during 1960–2007, containing approximately 80 economic and financial crises. In other words, the fast recovery of the U.S. can be an important reason for its smoother domestic output gap compared with most trading countries. Second, it can be found that the output gap in the U.S. is smoother than each trading partner countries. Therefore, it is not surprising that the foreign output gap weighted by 13 trading countries shows greater volatility than that of the U.S. as shown in **Fig 2**. In addition, the changing trend of the technology drag on variable is crosscurrent consistent with core inflation, but it is less volatile than is the latter. As for the other three control variables federal funds rate, U.S. dollar index and nonfarm labor productivity, the federal funds rate and core inflation have shown a common trend to some extent. It is clear that before 2009, the federal funds rate changed before core inflation and was less volatile. After 2009, the federal funds rate is basically maintained at 0 and core inflation is also fluctuating around 2%. The trend of the U.S. dollar index and the core inflation have become certain trend. That is, changes in the U.S. dollar index will drive the opposite direction of inflation. This may be related to the decline in domestic commodity prices caused by rising exchange rates. The nonfarm labor productivity and inflation have followed a similar trend. This is consistent with the traditional theory that the growth rate of labor productivity determines the growth rate of real wages, which further affects the inflation. As for core inflation, except for 2005–2008, inflation is below the target of 2% during the rest of the study period. The information presented in **Fig 2** suggests that globalization and technological factors may be important factors that cannot be ignored in the study of the dynamics of core inflation in the United States.

## 4. Empirical results

### 4.1 Econometric issues

In this paper, eight time series of inflation, inflation expectations, lagged inflation, domestic output gap, and foreign output gap, drag of technology variables, federal funds rate and U.S. dollar index are taken as empirical research variables.

Because macroeconomic variables are mostly non-stationary, econometric models based on non-stationary sequences can easily lead to "pseudo-regression". Thus, before constructing an econometric model for an in-depth empirical analysis, the first step is to test the stability of the variable sequence, the unit root test. The most common unit root test methods include the ADF test [56] and the PP test [57].

The linear unit root test method implies an assumption that the data generation process (DGP) has no structural breaks. However, in the real economy, severe exogenous shocks, such as institutional changes, changes in macroeconomic policies, and financial crises, may cause the data generation process of variable sequences to occur structural mutations. If this structure mutation is not considered, using a traditional unit root test, it is easy to take a structural mutation (including mutations and trends in level values). The potential sudden change in the smooth process of the potential trend is misjudged as a random trend non-stationary process.

To compensate for the deficiencies of the traditional unit root test, Perron [58] pioneered the unit root test of structural breaks. However, Perron's research on the selection of structural mutation points is based on prior knowledge and data mining of variable sequences. It is assumed that the structure mutation point is known (exogenous), which may lead to the unit root test on the null hypothesis excessive rejection. Subsequently, Zivot and Andrews [59–61] proposed a unit root test for structural mutations—the ZA test. The method abandons the a priori hypothesis of the structure mutation point, internalizes the structure mutation point, and makes the data sequence itself determine its structure mutation point.

In addition, the real variables at the current period in Model (3) are likely to be correlated with the contemporaneous random disturbance variable. Specifically, since inflation expectations are based on forecasts from all relevant information in and after t, the stochastic factors affecting the spot inflation rate (such as supply shocks due to changes in international oil prices) are likely to affect inflation expectations. At the same time, according to the standard macroeconomic analysis framework (such as Stock & Watson [62]), random factors affecting the spot inflation rate are also likely to affect the immediate output gap (both domestic and foreign). Therefore, we use either instrumental variables (IVs) or the GMM estimator to estimate Model (3) and eliminate the endogeneity problem. The baseline IV set includes lags of inflation and inflation expectations in the regression model, the 1–2 lagged terms of the domestic and foreign output gap, the technology variable, and the growth rate of M2 in the U.S. [63]. The optimal lag order in the model is specified by Akaike information criteria (AIC). The rationality of instrumental variable selection is further determined by Hansen's J test [64], which assumes that all instrumental variables are exogenous, and if the null hypothesis is not rejected, the choice of instrumental variables is relatively reasonable.

### 4.2 Baseline results

Taking into account the impact of external shocks may lead to structural changes in the DGP. To more accurately discriminate the stability of the above variables, we using the structure of the unit root test—ZA test. The result of the ZA unit root test for model (3) is shown in **Table 1**.

**Table 1:** Time series variables—ZA Unit root test results.

Variable	Lag Length	t-Statistic	P-Value	Break Date
*π*_*t*_	7	-3.9731	0.1650	2008:Q2
*E*_*t*_*π*_*t*+1_	0	-4.9432	0.0103	2008:Q2
*π*_*t*−1_	7	-3.8495	0.2145	2008:Q3
$y_{t}^{d}$	1	-2.1679	0.9668	2006:Q1
$y_{t}^{f}$	8	-4.6416	0.0291	2006:Q1
*tech*_*t*_	3	-5.4146	<0.01	2008:Q2
*γ*_*t*_	10	-4.918	0.0120	2011:Q3
*USDλ*_*t*_	6	-3.969	0.1662	2014:Q1
*NLP*_*t*_	8	-5.2414	0.69	2009:Q2

Optimal lag length is automatic specified based on AIC with a maximum eleven lags. Break selection by minimize Dickey-Fuller t-statistic. Break date correspond to the break point at which the maximum test statistic is achieved.

As can be seen from the results in **Table 1**, asymptotic one-sided p-values [65] for four variables’ (inflation expectations, foreign real output gap, technical drag variables and federal funds rate) break test statistics are significant at different significance levels (5% and 1%). Other variables are not significant even at the 10% significance level. The break date statistics do not provide a uniform break point. Nonetheless, the structural break tests of interest, namely the foreign output gaps and the drag of technology variables, both have significance break data (2006:Q1 and 2008:Q2). The federal funds rate has a significance break data (2011:Q3).The domestic output gap, U.S. dollar index and nonfarm labor productivity’s break points (2006:Q1, 2014:Q1 and 2009:Q2) are not significant. In addition, inflation expectations has a significance break data (2008:Q2).

Since the structural break tests with unknown points fail to identify a clear break point for the underlying model, we need to produce additional empirical evidence based on a more comprehensive set of formal parameter constancy tests in support of the identified break points. Therefore, we define dummy variables based on change points in 2006:Q1,2008:Q2 and2011:Q3 as the structural break in output gap measures and technical drag variables and to alleviate the associated collinearity induced by the multiplicative dummy variables, we use the following model to carry out dummy tests for each individual break point:

$$\begin{array}{c} \pi_{t}=c+\gamma_{e}E_{t}\pi_{t+1}+\gamma_{b}\pi_{t-1}+\delta_{d}y_{t}^{d}+\delta_{f}y_{t}^{f}+\phi_{g}tech_{t}\\ +\delta_{d}^{\prime }y_{t}^{d}d_{t}+\delta_{f}^{\prime }y_{t}^{f}d_{t}+\phi_{g}^{\prime }tech_{t}d_{t}+X_{t}\beta +\eta_{t} \end{array}$$ (4)

where *d*_*t*_ denotes the dummy variable with zero before the break point and unity otherwise. $\delta_{d}^{\prime }$, $\delta_{f}^{\prime }$, $\phi_{g}^{\prime }$ are the coefficients of domestic output gap, foreign output gap, drag of technology variables that take into account the dummy variables. If the statistical results show that $\delta_{d}^{\prime }$, $\delta_{f}^{\prime }$, $\phi_{g}^{\prime }$ is significantly not equal to zero, it indicates that those three variables have changed significantly before and after at the structural break point.

The results for the dummy tests with the three possible break points are reported in **Table 2** using the sample 1999:Q1–2016:Q4. Based on the break point of the 2006:Q1,2008:Q2 and 2011:Q3 regression estimate, both individual coefficient tests and the joint significance test are statistically significant, which provides clear evidence that the coefficients on domestic output gap, foreign output gap, and technical drag variables experience significant structural change in the three break points periods. It notices that except for foreign output gap and technical drag variables in 2008, the three variables have the same significance at the 1% level in all break points sample period. The results for the dummy tests also cannot help identify a clear break point for the underlying model. As already noted in **Fig 1**, the core inflation rate remains above 2% in 2006 while it goes below the 2% level in 2008, at which time the financial crisis erupted. Combining the dummy tests and real economic facts, we choose the structural break point in 2008:Q2 in this paper, while another possible break points (2006:Q1 and 2011:Q3) will be examined in the robustness analysis.

**Table 2:** Results of dummy tests for model 4.

Break Point	2006:Q1	2008:Q2	2011:Q3
*γ*_*e*_	0.15 (0.11)	-0.01 (0.13)	0.02* (0.1)
*γ*_*b*_	0.39*** (0.09)	0.53*** (0.1)	0.54*** (0.09)
*δ*_*d*_	-0.02 (0.03)	-0.003 (0.04)	-0.004 (0.04)
*δ*_*f*_	-0.01 (0.02)	-0.005 (0.03)	-0.002 (0.03)
*φ*_*g*_	-0.12*** (0.01)	-0.12*** (0.01)	-0.12*** (0.01)
$\delta_{d}^{\prime }$	0.15*** (0.13)	-0.14 (0.25)	0.07*** (0.02)
$\delta_{f}^{\prime }$	0.02*** (0.04)	-0.03** (0.01)	-0.12*** (0.02)
$\varphi_{g}^{\prime }$	-0.01** (0.004)	-0.01** (0.004)	-0.23*** (0.007)
*β*_*r*_	0.01 (0.04)	0.01 (0.03)	-0.0003 (0.02)
*β*_*USDX*_	-0.02*** (0.01)	-0.02** (0.01)	-0.02** (0.01)
*β*_*NLP*_	0.01 (0.02)	-0.01 (0.02)	-0.01 (0.02)
Joint Test	33.1***	0.29**	0.0009***

The estimated equation is given by model 4 over the sample period from 1999:Q1 to 2016:Q4 prior to lag adjustment. “Joint Test” denotes the p-value for a joint significance test (F-statistic) for the coefficients on all dummy variables.

*, **, and *** denote statistical significance at the 10, 5, and 1 percent levels, respectively.

Overall, the results of the structural break tests confirm that the U.S. experienced a significant structural break during the financial crisis of 2008, and the evidence of change relates to the domestic output gap, the foreign output gap, and technical drag, in addition to components in the extended hybrid NKPC model. Now we have a structural break point, based on which we can investigate the nature of changes in the U.S.’s inflation dynamics and compare the impact of globalization and technology on inflation dynamics over different sample periods when the break in the underlying coefficients is recognized near the 2008 financial crisis period. In what follows, we will take the break date pertaining to the coefficient on the output gap and technical drag (i.e., 2008: Q2) as the benchmark time to split the sample to research the coefficient change which our interest variables at pre- and post- the structural break point.

Based on the above description, **Table 3** reports the estimation (GMM) results for model (3) over the whole sample, pre- and post-2008:Q2,where the first 2–8 rows are the estimated value and the Newey–West (fixed bandwidth) HAC robust standard errors of the core parameters in the model. The ninth row reports the Hansen’s J-test (1982) corresponding p-values. The tenth row is the weak IV refer to weak instrumental variables (IV) test. According to [66], validity testing of instrumental variables includes correlation testing and exogenous testing. Therefore, we have added the results of Hansen’s J-test with the original hypothesis that all instrumental variables are exogenous and the weak instrumental variables test of [66]. Specifically, the P-J values in **Table 3** are larger than 10%, meaning that we should reject the null hypothesis. The F values are larger than 10 (the critical values are provided in [66] in most cases, thus suggesting that IVs are comparatively valid for our empirical analysis. The second to last column shows the variables’ Variance Inflation Factor (VIF), which tests whether the variables have multicollinearity, and the goodness-of-fit statistic R^2^ is reported in the last line. The diagnostic test statistics in **Table 3** indicate that the max VIF value is far below 10, which means that Eq (3) does not have multiple collinearity problems and the Eq (3) has a high overall fit.

**Table 3:** (GMM) Estimation Results of the inflation dynamics for the United States.

	(1) 1999: Q1–2016: Q4	(2) Pre-2008: Q2	(3) Post-2008: Q2
*γ*_*e*_	0.03 (0.08)	0.06 (0.21)	0.27 (0.18)
*γ*_*b*_	0.38*** (0.06)	0.51*** (0.14)	0.16** (0.08)
*δ*_*d*_	-0.03*** (0.01)	0.009 (0.05)	-0.02** (0.01)
*δ*_*f*_	0.003 (0.01)	-0.01 (0.05)	0.02** (0.02)
*φ*_*g*_	-0.11*** (0.01)	-0.12*** (0.01)	-0.14*** (0.01)
*β*_*r*_	0.02*** (0.01)	-0.002 (0.02)	0.04 (0.05)
*β*_*USDX*_	-0.008*** (0.003)	-0.03** (0.01)	0.004 (0.004)
*β*_*NLP*_	-0.02** (0.01)	-0.02 (0.02)	0.01 (0.01)
p-J	0.38	0.95	0.62
weak IV	13.91	13.91	13.91
Max VIF	2.52	3.52	4.23
*R*^2^	0.93	0.92	0.94

The estimated equation is given by model (3).The sample range is from 1999:Q1 to 2016:Q4; Instrumental variables set used in the regression model consists of two lags of the domestic output gaps, foreign output gaps and technical variables, and 1 year-on-year growth rate of M2 in the U.S.; p-J and weak IV refers to the p-value of the J-test in [64] (the original hypothesis is that all instrumental variables are exogenous) and the weak instrumental variables test in [66] (critical values for the weak IV test are provided in [66]); The max VIF is generally no more than 10; the Newey–West standard deviation of robustness (HAC, fixed bandwidth) is reported in parentheses

*, **, and *** denote statistical significance at the 10, 5, and 1 percent levels, respectively.

The baseline results reported in groups (1), (2), and (3) in **Table 3** reveal significant changes in the impact of the domestic output gaps, foreign output gaps, and technical drag variables on U.S. inflation over different sample periods. Specifically, group (1) shows that, if the structural break is neglected, the foreign output gap is not significantly, but the domestic output gap and technical drag drive inflation significantly, with coefficient estimates of -0.03 and -0.11, respectively, at the 1% significance level. The driving force of domestic output gap on inflation rate does not match the traditional theory. This result shows that, under the same conditions, a one percentage point increase in the domestic output gap and technology drag on inflation change will significantly reduce the domestic inflation rate, by 0.03 and 0.11 percentage points, respectively. In other words, the development status of technological innovation will significantly affect the trend of the domestic price change rate in the U.S. while globalization did not produce such an effect during the sample period. Perhaps the most surprising finding of the regression results is that the coefficient of the technical variable is larger than the coefficient of the domestic output gap. This means that, in the past twenty years or so, technological innovation has significantly exceeded the impact of the domestic output gap over the inflation dynamics in the United States. This result confirms the original intention of this paper, which is that, when discussing the inflation changes, technology is often an overlooked factor, but it may be an influential factor. As for the three control variables, the federal funds rate and U.S. dollar index have significant positive effects on inflation at the significance level of 1%. The impact of the nonfarm labor productivity on inflation is significant at the significance level of 5%.

However, when considering structural break, the results have changed. Group (2) and (3) prove that the impacts of the domestic output gap, the foreign output gap, and technology on inflation change during the period around 2008. The size and significance level of the domestic output gap coefficients improves from the insignificant 0.009 pre-2008 to -0.02 at the significant level of 5% post-2008. Meanwhile, the coefficient of the foreign output gap improves from the insignificant and negative value of -0.01 to significant and positive value of 0.02. This result shows that the impact of globalization, which is expressed as the output gap of foreign countries on U.S. inflation, reverses after 2008. In addition, the coefficient of the technical drag rises from a significant and negative value of -0.12 to -0.14. This shows that the impact of technological innovation on U.S. inflation has slightly increased after 2008. The coefficients of three control variables changed around 2008. The U.S. dollar index has a significant negative effect on domestic inflation in the pre-2008 period, while the other control variables are not significant in these two periods.

The results indicate that the domestic output gap and foreign output gap play a same size role in the hybrid NKPC of the U.S. after 2008, although the coefficient estimate of the two variables have opposite sign and they all become significant. One possible explanation is that the United States has strengthened cooperation with countries and regions in various economies in response to the financial crisis that occurred in 2008, making the United States more affected by changes in the world economy. This may lead to the impact of foreign output gap variable on U.S. domestic inflation to become significant. Besides, the results of the study also show that the impact of technology on inflation has been enhanced since 2008. This indicates that technology’s role increases in production processes as costs continue to fall. The amount of technology used in production processes has more than doubled since the late 1990s, from $0.08 per $1 of output to $0.20 in real terms. The rate of technology inputs in the production process is gradually accelerating [9]. Therefore, it can be considered that technology will continue to have an impact on inflation, and we have no reason to believe that this trend will reverse any time soon.

### 4.3 Robustness assessments

To assess the robustness of the baseline finding that the impact of globalization and technology on inflation dynamics changes before and after 2008 in the U.S., we conduct two sets of sensitivity studies. First, we investigate whether the finding is robust to slightly different break points of 2006: Q1 and 2011: Q3, which are shown in **Table 1**. Second, we assess the robustness of the baseline finding by dropping the foreign output gap and the technical drag variable from the extended version of the hybrid NKPC model. If the foreign and technology economic slack does not exert any influence on the sensitivity of inflation to the domestic output gap, the coefficient estimates on the domestic output gap in models both with and without foreign output gap and the technology variable should be similar.

**Table 4** reports the results for the first sensitivity study. The results for break points being 2006: Q1 and 2011: Q3 provide a scenario similar to the baseline finding. For the breakpoint of 2006: Q1, the coefficient of the foreign output gap is negative and insignificant at the post-2006 period, whereas it is significant (0.02) at the 5% significance level post-2008. For the breakpoint of 2011: Q3, the coefficient of the foreign output gap post-2011 is significantly different from the coefficient post-2008.The estimates for other variables in the model generally agree with expectations. The regression results also support the baseline conclusions.

**Table 4:** Robustness: Alternative break dates.

Sample	Baseline Estimates								Diagnostic Tests		
	*γ*_*e*_	*γ*_*b*_	*δ*_*d*_	*δ*_*f*_	*ϕ*_*g*_	*β*_*r*_	*β*_*USDX*_	*β*_*NLP*_	p-J	Max VIF	*R*^2^
Break 2006:Q1											
Pre-2006	0.36*** (0.11)	0.52*** (0.11)	0.02 (0.04)	-0.02 (0.03)	-0.11*** (0.02)	0.05** (0.03)	-0.03*** (0.01)	-0.01 (0.02)	0.38	4.17	0.80
Post-2006	0.26 (0.17)	0.22*** (0.05)	-0.001 (0.01)	-0.02 (0.02)	-0.13*** (0.01)	0.03 (0.03)	0.0001 (0.004)	0.02 (0.01)	0.35	2.91	0.96
Break 2011:Q3											
Pre-2011	-0.09 (0.26)	0.52*** (0.13)	-0.05* (0.03)	0.03 (0.05)	-0.11*** (0.01)	0.02* (0.01)	-0.01 (0.01)	-0.01 (0.01)	0.91	2.08	0.92
Post-2011	-0.1 (0.11)	0.02 (0.05)	-0.05*** (0.02)	0.02** (0.01)	-0.14*** (0.01)	-0.19** (0.1)	-0.002 (0.01)	0.01 (0.01)	0.17	3.47	0.99

The estimated equation is given by model (3).The sample range is from 1999:Q1 to 2016:Q4; Instrumental variables set used in the regression model consists of two lags of the domestic output gaps, foreign output gaps and technical variables, and 1 year-on-year growth rate of M2 in the U.S.; p-J and weak IV refers to the p-value of the J-test in [64] (the original hypothesis is that all instrumental variables are exogenous) and the weak instrumental variables test in [66] (critical values for the weak IV test are provided in [66]); The max VIF is generally no more than 10; the Newey–West standard deviation of robustness (HAC, fixed bandwidth) is reported in parentheses

*, **, and *** denote statistical significance at the 10, 5, and 1 percent levels, respectively.

**Table 5** summarizes the results for the second exercise sensitivity study. Groups (1), (2) and (3) provide the estimation results when foreign output gap and technology, foreign and technology are dropped from the hybrid NKPC model. For the full sample period of 1999–2016, we note that if the model establishment without the foreign output gap and technology variables, the point estimate of the domestic output gap does not change,. However, the sign of the coefficient changes, and the driving direction of the inflation rate is consistent with the traditional theory. When foreign output gap is not considered, the coefficient of domestic output gap is not significant. When adding the foreign output gap, the coefficient of domestic output gap turns significant. This shows that the technology variable plays an important role in model stability during the whole sample period and also has a larger point estimate. For the pre-2008 period, when the technology is not considered, the coefficient of domestic output gap is not significant, regardless of whether considering the globalization variable. When adding technology, the coefficients of technology and control variable of U.S. dollar index are both significant. This shows that, in the NKPC framework, technology is a key variable to explain U.S. inflation. However, it is clear that the technology and the foreign output gap both play significant roles in driving the U.S. inflation after 2008. Meanwhile, the technology plays a large role compared with the foreign output gap in driving domestic inflation in terms of the magnitude of the coefficients. These results agree with the conclusion that technology and globalization are important in affecting the U.S. dynamic mechanism of inflation after 2008.

**Table 5:** Robustness: Dropping domestic, foreign or technology variables.

	Baseline Estimates								Diagnostic Tests		
	*γ*_*e*_	*γ*_*b*_	*δ*_*d*_	*δ*_*f*_	*ϕ*_*g*_	*β*_*r*_	*β*_*USDX*_	*β*_*NLP*_	p-J	Max VIF	*R*^2^
(1) 1999:Q1–2016:Q4											
No Foreign, Technology	0.02 (0.07)	0.67*** (0.07)	0.03*** (0.01)			0.01 (0.02)	-0.01*** (0.004)	-0.03 (0.02)	0.51	3.12	0.8
No Foreign	0.04 (0.04)	0.38*** (0.04)	-0.01 (0.01)		-0.1*** (0.01)	0.02*** (0.01)	-0.01*** (0.003)	-0.02** (0.01)	0.24	2.85	0.93
No Technology	0.06 (0.2)	0.66*** (0.1)	0.03** (0.02)	-0.004 (0.03)		0.01 (0.02)	-0.01*** (0.003)	-0.02 (0.02)	0.49	4.17	0.8
(2) Per-2008:Q2											
No Foreign, Technology	0.19* (0.1)	0.46*** (0.15)	-0.03 (0.03)			0.03 (0.03)	0.004 (0.01)	-0.05* (0.03)	0.48	2.92	0.77
No Foreign	0.01 (0.06)	0.53*** (0.1)	-0.002 (0.02)		-0.12*** (0.01)	0.002 (0.01)	-0.02*** (0.01)	-0.01 (0.02)	0.93	3.11	0.91
No Technology	0.01 (0.35)	0.6*** (0.22)	-0.04 (0.07)	0.03 (0.07)		0.02 (0.03)	0.01 (0.02)	-0.05 (0.02)	0.4	4.6	0.77
(3) Post-2008:Q2											
No Foreign, Technology	0.08 (0.16)	0.63*** (0.1)	0.05*** (0.02)			0.03 (0.04)	-0.01 (0.01)	0.01 (0.03)	0.59	5.96	0.72
No Foreign	0.2*** (0.05)	0.12*** (0.05)	-0.02* (0.01)		-0.14*** (0.01)	0.01 (0.02)	0.01 (0.003)	0.01 (0.01)	0.69	6.04	0.96
No Technology	0.29 (0.48)	0.67*** (0.16)	0.05** (0.02)	-0.02 (0.07)		0.08 (0.13)	-0.01 (0.01)	0.01 (0.03)	0.73	2.67	0.67

‘No Foreign and Technology’ means that the regression excludes the foreign output gap and technology variable, and ‘No Foreign / Technology’ is defined analogously; Instrumental variables do not include the lag items corresponding to each dropped item. The max VIF is generally no more than 10; the Newey–West standard deviation of robustness (HAC, fixed bandwidth) is reported in parentheses.

*, **, and *** denote statistical significance at the 10, 5, and 1 percent levels, respectively.

## 5. Conclusions

The inflation rate in the U.S. has been running low for a long time. The percent change in the Consumer Price Index (CPI) over the five years ending in December 2017 was the lowest rate of price increase seen in half a century. Low inflation is a broad phenomenon that characterizes almost all the components of the CPI and countries. Globalization have been claimed to be the key factor driving inflation all the ways. However, this story is not fully convincing, and inflation may not have become globalized, as evinced by some observers. For example, the rigid structure of some economies may hinder the response of the pricing process to globalization.

This paper has shown that globalization and technology both contribute to low inflation in the United States. Our empirical investigations show that there is a significant structural change in 2008 in the extended NKPC model for the U.S., and we present three additional findings that add to the literature on U.S. low inflation dynamics. First, technology and globalization can explain the low inflation in the United States. As the empirical results show, whether in the total sample time or the segmented sample time interval, technology and globalization are almost all have significant explanatory. Second, judging from the changing trend of the impact of variables, the impact of the domestic and foreign output gap on domestic inflation is weakening, whereas the impact of technology on inflation is increasing. This finding indicates that we should take into account the developments in global economic performance and technology innovation trends in understanding the dynamic process of inflation. Third, globalization and technology have different effects on inflation in the United States. At present, technology appears to exert stronger influence on U.S. inflation compared with globalization. Therefore, studies that neglect the role of technology and only pay attention to globalization are not likely to accurately capture trends in inflation dynamics.

The issue of low inflation has attracted the attention of increasing numbers of scholars and central banks in various countries. When talking about the problem of low inflation, people often talk about the causes of some old clichés, such as globalization, oil prices, and commodities. We show that technology is another important and often overlooked factor that causes low inflation. The pace of technological innovation is likely to remain an obstacle to central banks’ goal of 2% inflation. The impact of technology and globalization on inflation remains an issue, in that those two factors are important reasons for the difficulties that central banks face in achieving their mandates, and policy-makers must continue to monitor them.
